# Supplementary material for: Role and mechanism of NCAPD3 in promoting malignant behaviors in gastric cancer
Source: Front Pharmacol. 2024 Apr 22;15:1341039. doi: 10.3389/fphar.2024.1341039 (PMC11070777; doi:10.3389/fphar.2024.1341039)
Supplement: Supplementary file 11 [file DataSheet2.ZIP › GSEA/Oncogenic signature/my_analysis.Gsea.1599461962066/PRC2_EED_UP.V1_DN.html]

Details for gene set PRC2\_EED\_UP.V1\_DN[GSEA]

|  || Dataset | filtered\_dataset.sample\_info.cls#WT\_versus\_NCAPD3\_MUT |
| Phenotype | sample\_info.cls#WT\_versus\_NCAPD3\_MUT |
| Upregulated in class | WT |
| GeneSet | PRC2\_EED\_UP.V1\_DN |
| Enrichment Score (ES) | 0.439081 |
| Normalized Enrichment Score (NES) | 1.6859739 |
| Nominal p-value | 0.023850085 |
| FDR q-value | 0.108831584 |
| FWER p-Value | 0.743 |
Table: GSEA Results Summary

  

Fig 1: Enrichment plot: PRC2\_EED\_UP.V1\_DN      
 Profile of the Running ES Score & Positions of GeneSet Members on the Rank Ordered List

  

| SYMBOL | TITLE | RANK IN GENE LIST | RANK METRIC SCORE | RUNNING ES | CORE ENRICHMENT || 1 | 64172 | OSGEPL1 | 6 | 1.152 | 0.1205 | Yes |
| 2 | 55727 | BTBD7 | 38 | 0.954 | 0.2016 | Yes |
| 3 | 56112 | PCDHGA3 | 107 | 0.781 | 0.2376 | Yes |
| 4 | 5098 | PCDHGC3 | 118 | 0.781 | 0.3151 | Yes |
| 5 | 64794 | DDX31 | 248 | 0.633 | 0.2912 | Yes |
| 6 | 2241 | FER | 304 | 0.588 | 0.3156 | Yes |
| 7 | 55697 | VAC14 | 329 | 0.576 | 0.3608 | Yes |
| 8 | 10818 | FRS2 | 363 | 0.558 | 0.3977 | Yes |
| 9 | 8737 | RIPK1 | 493 | 0.477 | 0.3569 | Yes |
| 10 | 84951 | TNS4 | 554 | 0.445 | 0.3621 | Yes |
| 11 | 6835 | SURF2 | 582 | 0.434 | 0.3898 | Yes |
| 12 | 23404 | EXOSC2 | 609 | 0.418 | 0.4164 | Yes |
| 13 | 4047 | LSS | 640 | 0.405 | 0.4388 | Yes |
| 14 | 10607 | TBL3 | 698 | 0.380 | 0.4391 | Yes |
| 15 | 2355 | FOSL2 | 1262 | -0.648 | 0.1060 | No |
Table: GSEA details [plain text format]

  

Fig 2: PRC2\_EED\_UP.V1\_DN      
 Blue-Pink O' Gram in the Space of the Analyzed GeneSet

  

Fig 3: PRC2\_EED\_UP.V1\_DN: Random ES distribution      
 Gene set null distribution of ES for **PRC2\_EED\_UP.V1\_DN**
